# Comparative Effects of *Capsicum annuum*-Derived Selenium Nanoparticles and Sodium Selenite on Reproductive Performance, Egg Quality, and Male Sexual Behavior in Japanese Quails

**DOI:** 10.3390/ani15233379

**Published:** 2025-11-21

**Authors:** Hanan Al-Khalaifah, Sania Satti, Rasha Alonaizan, Shabana Naz, Sajida Arooj, Azka Haseeb, Rifat Ullah Khan, Ala Abudabos

**Affiliations:** 1 Environment and Life Sciences Research Center, Kuwait Institute for Scientific Research, Safat 13109, Kuwait; 2 Department of Zoology, Government College University, Faisalabad 38000, Pakistan; saniasatti448@gmail.com (S.S.); sajidaarooj1@gmail.com (S.A.);; 3 Department of Zoology, College of Sciences, King Saud University, Riyadh 11451, Saudi Arabia; 4 Physiology Lab, College of Veterinary Sciences, Faculty of Animal Husbandry and Veterinary Sciences, The University of Agriculture, Peshawar 25100, Pakistan; rukhan@aup.edu.pk; 5 Department of Food and Animal Sciences, College of Agriculture, Tennessee State University, Nashville, TN 37209, USA

**Keywords:** Japanese quail, selenium nanoparticles, sodium selenite, reproductive performance, sexual behavior

## Abstract

The effects of selenium on poultry health and reproduction have been extensively studied using inorganic sodium selenite (SS). However, limited research has been done on organic selenium nanoparticles (Se-NPs). This study aimed to compare Se-NPs and SS on productivity, egg quality, reproductive performance, and male sexual behavior in Japanese quails. A total of 480 quails (8 weeks old) were assigned to 5 different experimental groups (96 birds in each group) with six replicates (pens) sex ratio 1 male: 3 females. One group was designated as control (fed by basal diet), whereas two groups were fed with different levels of Se-NPs (0.2 mg/kg and 0.4 mg/kg), and two groups with SS (0.2 mg/kg and 0.4 mg/kg). The trial lasted for 9 weeks. The highest productive performance was observed in quails supplemented with 0.4 mg/kg Se-NPs (*p* < 0.05). Quails supplemented with 0.2 mg/kg Se-NPs had the highest egg weight (*p* < 0.05). The highest shape and albumen index were identified in the group supplemented with 0.2 mg/kg Se-NPs (*p* < 0.05). However, the highest shell ratio, yolk ratio, yolk index, and Haugh unit were determined in the group supplemented with 0.4 mg/kg Se-NPs (*p* < 0.05). The group supplemented with 0.4 mg Se-NPs/kg had the highest fertility and hatchability, with the lowest embryo mortality. The group with 0.4 mg Se-NPs/kg exhibited the highest level of sexual behavior (wing flapping, waltzing, mounting, tidbitting, rear approach, treading). It was concluded that the supplementation of Se-NPs enhanced productive and reproductive performance, egg quality, and male sexual behavior compared to the supplementation of SS.

## Simple Summary

This study compared the effects of selenium nanoparticles (Se-NPs) and inorganic selenium (sodium selenite, SS) on productivity, egg quality, reproduction, and male sexual behavior in Japanese quails. A total of 480 quails were divided into five groups and fed diets containing either Se-NPs or SS at two levels (0.2 and 0.4 mg/kg) for nine weeks. Quails receiving 0.4 mg/kg Se-NPs showed the best production performance, fertility, hatchability, and sexual activity, while Se-NPs also improved egg quality traits compared to SS. Overall, Se-NPs were more effective than inorganic selenium in enhancing productive and reproductive performance in Japanese quails.

## 1. Introduction

Japanese quails (*Coturnix coturnix japonica*) are small, fast-growing birds widely used in agricultural research and commercially raised for their meat and high egg production. They typically begin laying eggs at around 6 weeks of age and can produce between 250 and 300 eggs annually [1]. Known for their adaptability to diverse environmental conditions and rapid growth rate, they serve as an excellent model for physiological and nutritional studies [2]. Japanese quails are among the most prolific egg-laying avian species, valued for their high production rate, early maturity, and economic efficiency in small- and large-scale poultry systems. Their eggs are rich in nutrients and have growing commercial demand due to their flavor and health benefits. Because of their short generation interval, high reproductive efficiency, and adaptability to intensive housing, Japanese quails are also widely used in genetic, physiological, and nutritional studies related to egg production and fertility.

Selenium (Se) plays a pivotal role in poultry nutrition, particularly in supporting reproductive performance and egg-laying efficiency [3,4]. It functions as an essential component of several selenoproteins and antioxidant enzymes, such as glutathione peroxidase, which safeguard cellular integrity against oxidative stress [5,6]. These beneficial effects are mainly due to its role in enhancing selenoprotein synthesis and antioxidant defenses. Adequate selenium intake enhances ovarian activity, egg formation, sperm viability, and hatchability, whereas selenium deficiency is often linked with poor fertility and reduced hatchability rates in poultry [7,8]. Therefore, understanding selenium’s biological functions is essential when evaluating its role in improving reproductive traits and egg production in Japanese quails.

Recent research has focused on optimizing the nutritional composition of animal diets, emphasizing nutrients efficacy, enhance bioavailability, and reduce environmental impact [9,10,11]. Nanotechnology has emerged as a promising approach for improving nutrient delivery and utilization at the molecular and atomic levels. In particular, nanoparticles of trace elements have been incorporated into poultry diets, influencing egg production rates, blood biochemical parameters, and antioxidant activity [12]. Among these, selenium nanoparticles (Se-NPs) are notable for their high bioavailability and low toxicity, making them an important trace mineral in animal nutrition [13].

Selenium (Se) is commonly added to poultry feed in two forms: organic (such as selenocysteine, selenomethionine, and their methylated derivatives) and inorganic (such as sodium selenite and sodium selenate) [14]. The organic form enhances Se deposition in tissues and eggs while reducing its excretion into the environment through feces. However, excessive supplementation can be harmful to animals [15]. Both forms of Se may lead to the production of toxic metabolites or increase vulnerability to oxidative stress when overused [16]. According to Ali et al. [17], Se-NPs offer several advantages over organic and inorganic forms, including higher absorption rates, larger surface area, and greater particle diffusion. Reda et al. [18] reported that Se-NPs can readily cross plasma membranes and interact with biological systems, thereby improving bioavailability. Their small particle size and large surface area enhance epithelial permeability and gastrointestinal absorption, often through the formation of stable nano-emulsion-like dispersions. In poultry, their supplementation positively influences various physiological functions, such as boosting immune responses, enhancing antioxidant defenses, and supporting metabolic activity. The improved bioavailability of selenium from Se-NPs contributes to enhanced antioxidant enzyme activity, stronger immunity, and overall better health outcomes in poultry [7,19].

In recent years, significant research progress has been made in developing nano-selenium as a functional feed additive in poultry production. Studies have demonstrated that Se-NPs not only improve growth and productivity but also enhance reproductive parameters such as fertility, sperm quality, and hatchability. The superior antioxidant potential of nano-selenium contributes to maintaining reproductive tissue integrity and hormone regulation, thereby promoting egg-laying performance.

Recently, the green synthesis of Se-NPs has gained attention as an eco-friendly, cost-effective, and non-toxic alternative to conventional chemical and physical synthesis methods. This approach utilizes plant extracts, microorganisms, or biopolymers as natural reducing and stabilizing agents, eliminating the need for hazardous chemicals and high energy inputs [3]. Plant-based green synthesis, in particular, offers a sustainable strategy to produce stable, biocompatible Se-NPs with enhanced biological activity due to the presence of natural phytochemicals that coat the nanoparticles [20]. Such biosynthesized Se-NPs have demonstrated superior antioxidant and antimicrobial potential, making them highly suitable for application in animal nutrition and feed biotechnology [21].

Se-NPs represent an effective selenium source for optimizing poultry growth performance compared to other selenium forms [22]. Studies have shown that Se-NPs significantly impact poultry production by increasing fertility and egg production [23,24]. Dietary supplementation with Se-NPs has been found to improve both egg production and selenium content in eggs. For example, layers fed with 0.3 mg/kg of Se-NPs showed similar enhancements [25]. These findings highlight the role of Se-NPs in boosting egg production and generating selenium-enriched eggs. Experimental studies also suggest that they enhance the activity of glutathione peroxidase an enzyme that prevents lipid peroxidation, maintains cell membrane flexibility, and improves sperm fertilization capability [26]. Furthermore, dietary Se-NP supplementation has been associated with increased sperm motility and count, along with reduced incidence of dead and abnormal sperm [27]. They also promote fertilization processes and prolong sperm survival in the vas deferens. Notably, they contribute to improved hatchability and embryonic development by mitigating oxidative stress caused by free radicals [28]. Despite strong evidence primarily from studies in chickens demonstrating the superior bioavailability and physiological benefits of Se-NPs, there remains a lack of information regarding their effects in quails. Based on the current findings related to Se-NPs and SS, it is hypothesized that supplementing quails with Se-NPs will significantly enhance their productive performance. Additionally, Se-NP supplementation is expected to improve egg quality parameters, increase fertility and hatchability rates, and lead to greater frequency and duration of courtship displays and mating behaviors in male quails.

## 2. Materials and Methods

### 2.1. Experimental Birds, Design and Diets

This experiment was conducted in accordance with the guidelines for the care and handling of laboratory animals established by Government College University, Faisalabad (GCUF). A total of 480 eight-week-old quails, with uniform body weight (185 ± 10 g) and production rate (70 ± 2%), were randomly assigned to five experimental groups, as outlined in Table 1, based on selenium supplementation levels recommended by Nassef et al. [29]. Each group comprised 96 birds, divided into six replicates of 16 birds per cage, considering as experimental unit, maintaining a consistent sex ratio of 1 male to 3 females (4 males and 12 females per cage) [30]. The experimental unit was the cage, housing 16 quails. Birds were kept in cages made of 7 mm square welded wire mesh, providing approximately 125 cm^2^ of floor space per bird (total cage floor area ≈ 2000 cm^2^; e.g., 40 × 50 cm), in accordance with standard welfare recommendations. Each cage was covered with a solid metal or plywood roof to minimize head injuries caused by startling, following established quail management practices [31]. Treatments were administered via daily oral gavage to ensure precise dosing based on body weight (mg/kg). Although effective, this method may produce a “bolus effect,” potentially affecting absorption, distribution, metabolism, and excretion in ways that differ from real-world exposure scenarios [32]. The main ingredients and nutritional composition of the basal diet are provided in Table 2, while Table 3 showed Se concentration in feed and excreta and apparent retention. The trial lasted nine weeks, including a one-week acclimation period followed by an eight-week treatment period. Environmental conditions were maintained according to Japanese quail husbandry standards: temperature was kept between 23 and 26 °C, and relative humidity between 65 and 75%, with a 16:8 h light–dark photoperiod and mechanical ventilation to ensure proper air quality. Birds had ad libitum access to feed and water formulated to meet their nutritional requirements ([33]; Table 3).

### 2.2. Synthesis of Selenium Nanoparticles

Fifteen kilograms of *Capsicum annuum* were thoroughly washed seven times with double-distilled water to eliminate any impurities. The cleaned samples were then chopped and subjected to air-drying in a shaded environment for 15 days. Following the drying process, the material was ground into a fine powder using a mechanical grinder. A 500 g portion of the powdered C. annuum was soaked in 1000 mL of ethanol. The resulting mixture was filtered, and the filtrate was concentrated using a rotary evaporator for 15 min. The concentrated extract was further dehydrated in a vacuum evaporator until a constant weight was achieved, yielding a dry extract cake. 50 mL of the C. annuum extract were then combined with 0.263 g of selenious acid (H_2_SeO_3_). The pH of the reaction mixture was adjusted to 5.4 using sodium hydroxide (NaOH) and allowed to react for 15 h, forming a red-colored solution. The mixture was subsequently subjected to centrifugation at 12,000 rpm for 4 min at room temperature. The precipitated Se-NPs were washed with deionized water and dried in vacuum desiccators to a constant weight [34]. The synthesized Se-NPs were characterized for their physicochemical properties using standard analytical techniques. X-ray diffraction (XRD) analysis confirmed the crystalline nature of Se-NPs, and Fourier-transform infrared spectroscopy (FTIR) was used to identify functional groups involved in nanoparticle stabilization. The average particle size ranged between 50 and 90 nm with a predominantly spherical morphology, indicating uniform synthesis and stability. The zeta potential of −28 mV suggested good colloidal stability, preventing aggregation and ensuring consistent biological activity.

### 2.3. Quantification of Selenium Concentration

The total selenium content in the synthesized SeNPs was quantified using inductively coupled plasma optical emission spectrometry (ICP–OES; Agilent Technologies, Santa Clara, CA, USA) after acid digestion. Approximately 10 mg of dried SeNPs were digested with 5 mL of concentrated nitric acid (HNO_3_) and 2 mL of hydrogen peroxide (H_2_O_2_) at 120 °C until a clear solution was obtained. The digested samples were diluted to 25 mL with deionized water and analyzed against selenium standards (0.1–10 mg/L). The selenium concentration (µg/mL) was calculated to ensure consistency and reproducibility among batches.

### 2.4. Estimation of Productive Performance

Feed intake was measured weekly. During weeks 9, 11, and 13, eggs were collected and weighed daily using a scale with a precision of 0.01 g. The production traits evaluated included Hen Day Egg Production (HDEP), egg mass, and feed conversion ratio (FCR). The following formulas were used to evaluate the respective parameters:

HDEP (%) = (Number of eggs produced ÷ Number of live hens × 100), Egg mass = (Number of eggs produced × Average egg weight), Feed Conversion Ratio (FCR) = (Total feed consumed ÷ Total egg mass).

### 2.5. Estimation of Egg Quality Parameters

At the end of the 9th, 11th, and 13th weeks, 60 eggs (10 eggs/replicate) from each group were gathered to calculate the egg quality index. The following parameters were studied and calculated:

$$Shape\;Index=\frac{Egg\;width\;(mm)\times 100}{Egg\;length\;(mm)}$$

Egg Shell Ratio: The egg shells were first cleaned and dried to ensure accurate weight measurements. The shell membrane was then carefully removed before weighing the shell. The egg shell ratio was calculated using the formula:

$$Egg\;shell\;ratio\;(\%)=\frac{Shell\;weight\;(g)\times 100}{Egg\;weight\;(g)}$$

Albumen Weight: Albumen weight was calculated as:

Albumen Weight = Egg Weight − (Yolk Weight + Shell Weight)

Yolk Ratio and Albumen Ratio: To measure yolk and albumen weight, the egg was carefully cracked, and the yolk was separated from the albumen. Each component was weighed separately on a scale, ensuring the scale was tared before each measurement. These ratios were calculated as the percentage of total egg weight:

$$Yolk\;ratio\;(\%)=\frac{Yolk\;weight\;(g)\times 100}{Egg\;weight\;(g)}$$

$$Albumen\;ratio\;(\%)=\frac{Albumen\;weight\;(g)\times 100}{Egg\;weight\;(g)}$$

Yolk Index (%): This was calculated using the formula:

$$Yolk\;index\;(\%)=\frac{Yolk\;height\;[mm]\times 100}{Yolk\;Diameter\;[mm]}$$

Albumen Index: The albumen index was determined using the formula:

$$Albumen\;index\;(\%)=\frac{Albumen\;height\;[mm]}{Albumen\;width\;[mm]}$$

Haugh Units: To calculate Haugh units, the height of the thick albumen was measured after cracking the egg on a flat surface. The formula used was [35]:

Haugh Units = 100 × log_10_ (H − 1.7 × W^0.37^ + 7.57)

### 2.6. Estimation of Reproductive Performance

During the experimental period, egg collection was conducted during the 9th, 11th, and 13th weeks of age to ensure consistent and reliable egg production. A total of 360 eggs per treatment group (60 eggs/replicate) were collected over one week, followed by collection in alternate weeks. The eggs were collected twice a day, preferably in the morning and late afternoon, to minimize contamination. Each egg was carefully inspected for cracks before being placed in clean, dry containers. To maintain freshness and prevent early embryonic development, the eggs were stored in a refrigerated cooler at 50 °F to 60 °F (10 °C to 15 °C) with 70% to 80% relative humidity for a short period (7 days). At the end of each collection week (i.e., the 9th, 11th, and 13th weeks), all stored eggs were incubated simultaneously at a single time point to ensure consistency across all treatment groups.

### 2.7. Incubation and Hatching

The eggs were incubated in an automated incubator (EYELA EYELATRON FLI-160, Shanghai, China) at a temperature of 37.5 °C (99.5 °F) and 60% relative humidity. Eggs were automatically turned every two hours (approximately 12 times per day) to ensure proper embryonic development and prevent adhesion of the embryo to the shell membranes. The eggs remained in the incubator between Embryonic Day 0 (ED0) to Embryonic Day 14 (ED14). Three days before hatching, on Embryonic Day 15 (ED15), the eggs were transferred to the hatcher. In the hatcher, the temperature was set to 36.7 °C (98 °F) and the humidity was increased to 70%.

Fertility was assessed by breaking all the eggs at the end of the eighteen-day incubation period. Fertility, hatchability, and embryo mortality percentages were calculated using the following equations [36].

Fertility percentage (%) = (Number of fertile eggs × 100)/Total number of eggs(1)

Hatchability of fertile eggs (%) = (Number of hatched chicks/Total number of fertile eggs) × 100(2)

Embryo mortality (%) = (Number of dead embryos) × 100/Number of fertile eggs(3)

### 2.8. Sexual Behavior

Sexual activity was continuously monitored over a period of three alternating weeks: the 9th, 11th, and 13th weeks of age. This timeframe was chosen as it likely represents the optimal period for observing sexual maturity and mating behaviors in quails. Mating activity was recorded through direct visual observation by trained human observers, rather than automated camera systems. Observers conducted continuous visual scanning for two hours daily, three days a week, with sessions divided into morning (06:00–12:00) and afternoon (12:00–18:00) slots. Each observation session lasted for one hour, with alternating rotations between morning and afternoon slots. The specific observation schedule was as follows: 06:00–07:00 and 12:00–13:00 on day one; 08:00–09:00 and 14:00–15:00 on day two; and so on for the remaining weeks. During each observation hour, the time was divided into 5 min intervals. Within each interval, all males in each replicate were systematically scanned to ensure thorough monitoring. Observations were recorded with precision, and male behaviors were noted during each scan. The specific behaviors observed and their frequencies were recorded as follows [24]:

Wing flapping: The male raises his wings above the level of his back and flaps them.
Waltzing: The male walks around the hen and extends his wings away from her.
Mounting: The male climbs onto the hen’s back and uses his claws to grab her wing feather.
Tidbitting: The male invites their mates by simulating the discovery of food in the litter
Rear approach: The male grasps the hen’s neck feathers with his beak.
Treading: The male takes small steps with his foot before mounting and making cloacal contact.

### 2.9. Statistical Analysis

Statistical analyses were performed using SPSS Statistics version 25 and Statistics version 8.1. The significance level was set at *p* < 0.05. Data were first assessed for normality using the Shapiro–Wilk test. Productive performance and egg quality parameters were analyzed using a linear mixed model, with treatment group and week as fixed factors and cage ID as a random factor, to account for repeated measures and the hierarchical structure of the data. All traits were evaluated at three time points: the 9th, 11th, and 13th weeks. Reproductive performance data were analyzed using a generalized linear mixed model (GLMM), considering treatment group and week as fixed effects and cage as a random effect. A binomial distribution and logit link function was used in the analysis reproductive performance as the response variable was binary (yes/no). Male sexual behavior was also analyzed using a GLMM, with Poisson regression applied due to the count-based nature of the data. Sexual behavior events were assumed to follow a Poisson distribution, and a log link function was used to model the rate of these events while accounting for random effects such as cage variation. A two-way ANOVA was additionally conducted to assess the main and interaction effects of diet and week on productive performance, egg quality, reproductive traits, and sexual behavior, assuming independence of observations where applicable. Tukey’s HSD test was used as a post hoc analysis to identify significant differences between treatment means.

## 3. Results

### 3.1. Effects of Se-NPs and SS Supplementation on Productive Performance of Japanese Quails

Table 4 illustrates the effects of different selenium sources over time on feed intake (FI), hen-day egg production (HDEP), egg weight, egg mass, and feed conversion ratio (FCR) in Japanese quails. As time progressed, FI, HDEP, egg weight, and egg mass increased significantly, while FCR decreased markedly (*p* < 0.001). The group receiving the highest Se-NP dose (0.4 mg/kg) exhibited the highest FI (21.81 g), HDEP (71.3%), egg mass (7.40 g), and the lowest FCR (2.94), compared to other treatments (*p* < 0.001). Conversely, the highest egg weight (10.40 g) was observed in the group receiving the lower Se-NP dose (*p* < 0.001). A significant dietary treatment × time interaction was observed for FI, HDEP, egg weight, egg mass, and FCR (*p* < 0.001). All productive performance parameters peaked in the 11th week for the high-dose Se-NP group (0.4 mg/kg) compared to the other groups (*p* < 0.001).

### 3.2. Impact of Se-NPs and SS Supplementation on the Egg Quality Indices of Japanese Quails

The impact of different selenium sources over time on various egg-quality indices in quails is shown in Table 5. Birds receiving a low dose of Se-NPs (0.2 mg/kg) exhibited significantly higher shape index (75.98%) and albumen index (10.17) compared to the control and other treatments (*p* < 0.001). Those given a high dose of Se-NPs (0.4 mg/kg) demonstrated significant increases in shell ratio (18.90%), yolk ratio (42.21%), yolk index (58.27), and Haugh unit (74.54) (*p* < 0.001). A significant dietary treatment × time interaction was observed for all egg-quality parameters except shell ratio (*p* > 0.05). All parameters peaked in week 11 in the high-dose Se-NP group (*p* < 0.001).

### 3.3. Influence of Se-NPs and SS Supplementation on the Reproductive Performance of Japanese Quails

The effects of different selenium treatments (Se-NPs and SS) over time on fertility %, hatchability % and embryo mortality % in quails are summarized in Table 6. Birds receiving Se-NPs at 0.2 mg/kg showed significantly higher fertility (80.00%) and hatchability (80.95%) compared to control and SS groups (*p* < 0.001). Those on Se-NPs at 0.4 mg/kg achieved the highest fertility (80.86%) and hatchability (82.38%) (*p* < 0.001). The percentage of embryo mortality was lowest in the Se-NPs 0.4 mg/kg group (17.62%) as compared to the control and other groups (*p* < 0.001). Binomial Logistic regression confirmed that Se-NPs 0.2 mg/kg (fertility: B = 1.325, *p* = 0.044; hatchability: B = 1.476, *p* = 0.016) and 0.4 mg/kg (fertility: B = 2.093, *p* = 0.011; hatchability: B = 2.064, *p* = 0.004) significantly increased odds of reproductive success, whereas SS treatments were non-significant, and Weeks had no effect (*p* ≥ 0.819). These findings demonstrate that Se-NP supplementation, particularly at 0.4 mg/kg, significantly enhances quail fertility and hatchability, while sodium selenite and time have little impact.

### 3.4. Effects of Se-NPs and SS Supplementation on the Sexual Behavior Frequencies of Male Japanese Quails

The impact of different selenium sources over time on male sexual behavior in quails is shown in Table 7. Males supplemented with a high dose of Se-NPs (0.4 mg/kg) exhibited the highest frequencies of wing flapping (88.17), waltzing (13.33), mounting (66.22), tidbitting (3.67), rear approach (78.56), and treading (86.94) compared to other groups (*p* < 0.001). A significant dietary treatment × time interaction was observed for wing flapping, tidbitting, and rear approach (*p* < 0.05), but not for waltzing, mounting, or treading (*p* > 0.05). All behavioral frequencies peaked in week 11 in the high-dose Se-NP group (*p* < 0.001).

### 3.5. Poisson Regression Analysis of the Impact of Se-NPs and SS Supplementation on Male Sexual Behavior in Quails

The Poisson regression analysis assessed the effects of low and high doses of Se-NPs and SS at Weeks 9, 11, and 13 on multiple behavioral traits in quails, using the control group as the baseline for comparison (Table 8). Regarding wing flapping behavior, quails supplemented with Se-NPs (0.2 mg/kg) exhibited significantly higher rates of this behavior (incidence rate ratio = 1.34, *p* < 0.001), and Se-NPs (0.4 mg/kg) also showed significantly higher rates of wing flapping (incidence rate ratio = 1.46, *p* < 0.001). The SS (0.2 mg/kg) group showed a modest but statistically significant increase in wing flapping (incidence rate ratio = 1.11, *p* < 0.001), while the SS (0.4 mg/kg) group did not show a significant difference compared to the control group (*p* = 0.124). Time, measured in weeks, did not have a significant effect on wing flapping behavior (*p* = 0.388), suggesting that the observed differences were primarily due to selenium supplementation rather than the passage of time.

In terms of waltzing behavior, Se-NPs supplementation had a significant effect. Se-NPs (0.2 mg/kg) significantly increased the likelihood of waltzing (incidence rate ratio = 1.37; *p* < 0.001), and Se-NPs (0.4 mg/kg) resulted in an even greater significant increase (incidence rate ratio = 1.66; *p* < 0.001). In contrast, SS supplementation did not significantly affect waltzing behavior (SS 0.2 mg/kg: incidence rate ratio = 1.04; *p* = 0.294; SS 0.4 mg/kg: incidence rate ratio = 1.00; *p* = 1.000). Additionally, time (Weeks) had a significant negative effect on waltzing behavior, with the likelihood of waltzing decreasing with each additional week (incidence rate ratio = 0.97; *p* < 0.001).

The analysis of mounting behavior demonstrated significant effects of selenium supplementation. Quails supplemented with Se-NPs (0.2 mg/kg) showed a significantly higher likelihood of mounting behavior (incidence rate ratio = 1.32; *p* < 0.001), and the Se-NPs (0.4 mg/kg) group also showed a significant increase (incidence rate ratio = 1.36; *p* < 0.001). Supplementation with SS also resulted in statistically significant increases. The SS (0.2 mg/kg) group showed a significant increase (incidence rate ratio = 1.05; *p* < 0.001), and the SS (0.4 mg/kg) group exhibited a significant increase as well (incidence rate ratio = 1.03; *p* = 0.039). Additionally, time (Weeks) had a significant negative effect on mounting behavior, with the likelihood decreasing over time (incidence rate ratio = 0.99; *p* = 0.017).

The most pronounced effects were observed in tidbitting behavior. Quails in the Se-NPs (0.2 mg/kg) and Se-NPs (0.4 mg/kg) groups exhibited significantly higher odds of displaying the behavior (incidence rate ratios = 3.33 and 3.66, respectively; *p* < 0.001 for both). Both SS-supplemented groups—SS (0.2 mg/kg) and SS (0.4 mg/kg)—also showed statistically significant increases (incidence rate ratio = 1.33; *p* = 0.006), although the magnitude of effect was smaller compared to Se-NPs groups. Time (Weeks) had no significant effect on tidbitting behavior (*p* = 1.000).

In the case of rear approach behavior, both Se-NPs-supplemented groups showed significant increases in the likelihood of this behavior compared to the control group. Quails in the Se-NPs (0.2 mg/kg) group had a significantly higher incidence rate ratio of 1.33 (*p* < 0.001), and those receiving Se-NPs (0.4 mg/kg) also showed a significant increase (incidence rate ratio = 1.37; *p* < 0.001). The SS-supplemented groups also exhibited significant increases—SS (0.2 mg/kg): incidence rate ratio = 1.08 (*p* < 0.001); SS (0.4 mg/kg): incidence rate ratio = 1.05 (*p* < 0.001). Time (Weeks) had a significant positive effect on rear approach behavior, with the likelihood increasing gradually over time (incidence rate ratio = 1.01; *p* < 0.001).

For treading behavior, Se-NPs and SS supplementation had a significant effect. Quails in the Se-NPs (0.2 mg/kg) group showed a significant increase (incidence rate ratio = 1.26; *p* < 0.001), and those in the Se-NPs (0.4 mg/kg) group also exhibited a significant increase (incidence rate ratio = 1.34; *p* < 0.001). The SS (0.2 mg/kg) group showed a significant increase (incidence rate ratio = 1.10; *p* < 0.001), as did the SS (0.4 mg/kg) group (incidence rate ratio = 1.08; *p* < 0.001). Time (Weeks) had a significant negative effect on treading behavior, with the likelihood decreasing over time (incidence rate ratio = 0.99; *p* = 0.004).

## 4. Discussion

In the present study, a week-wise evaluation revealed that both Se-NPs and SS significantly influenced the productive performance of Japanese quails, including feed intake, egg production, egg weight, egg mass, and feed conversion ratio (FCR) throughout the experimental period. The improved feed intake observed in quails supplemented with Se-NPs (0.4 mg/kg) is likely due to their high bioavailability, which enhances antioxidant enzyme activity and promotes gut health [7]. Such modulation enhances nutrient absorption and strengthens systemic immune responses, contributing to improved overall health and productivity in quails [37,38]. The observed increases in egg weight and egg mass in the Se-NPs group may be explained by the nanoparticles’ positive effects on ovarian function, potentially resulting in the formation of larger ova and, consequently, larger eggs. It is well established that poultry allocate a portion of their body weight to egg formation, which directly influences egg size at oviposition [39]. Enhanced productive performance is further supported by selenium’s role as an essential co-factor for the enzyme 5′-deiodinase [40], which catalyzes the conversion of inactive thyroxine (T4) to its biologically active form, triiodothyronine (T3). T3 plays a vital role in regulating growth, metabolism, muscle development, and ovarian function [41]. From a broader physiological perspective, these findings suggest that selenium status modulates key endocrine pathways involved in reproduction and growth. This regulatory mechanism enables the allocation of resources among maintenance, growth, and reproduction, particularly under physiological or environmental stress highlighting selenium’s importance beyond its antioxidant functions [42]. When comparing FCR across studies, it is important to consider that differences may result from variations in diet composition, genetic strain, management practices, environmental conditions, and bird age or sex [43,44].

In this study, egg quality parameters such as shape index and albumen index were significantly higher in quails receiving a low dose of Se-NPs compared to other groups. The increased shape index likely reflects geometric changes in the egg, particularly in the length-to-width ratio, rather than improvements in shell strength, as shape index is primarily determined by these dimensions [45]. While protoporphyrin IX pigmentation may contribute to shell strength [46], it does not directly affect egg shape. Instead, shell pigments mainly influence membrane synthesis through epithelial secretions, improving shell integrity and coloration rather than morphology [47]. Strengthening the shell membrane reduces egg breakage, which is critical for reproductive success by minimizing embryonic loss [48]. The increase in albumen index suggests that Se-NPs enhance moisture retention and protein content in the albumen. Since albumen quality is essential for nourishing and protecting the developing embryo, its preservation is vital. Physiological stress often reduces both pigmentation and albumen quality by causing oxidative damage, which leads to moisture loss and protein degradation [49]. The antioxidant properties of Se-NPs help mitigate this damage, preserving egg freshness and creating optimal conditions for embryonic development [50]. Moreover, the highest values for shell ratio, yolk ratio, yolk index, and Haugh unit were observed in quails treated with a higher dose of Se-NPs. These improvements in both shell strength and internal egg quality are likely due to increased levels of ovomucin a structural protein responsible for maintaining albumen thickness and viscosity, thereby protecting the yolk and embryo [51]. Oxidative degradation of ovomucin can occur when oxygen enters through eggshell pores, disrupting the lysozyme-ovomucin complex and reducing albumen thickness. Se-NPs help prevent this degradation by preserving structural proteins within the egg, highlighting a direct link between selenium’s antioxidant role and reproductive physiology. This preservation may also positively influence hatchability and chick viability, underscoring selenium’s role in embryonic development beyond basic nutrition [50]. Additionally, increased selenium deposition in eggs supports eggshell synthesis and facilitates selenium transfer to the embryo an essential factor in enhancing antioxidant defenses and promoting healthy growth [52].

In this study, a week-by-week comparison revealed that the Se-NP (0.4 mg/kg) group consistently demonstrated the highest reproductive performance in Japanese quails, with significantly greater fertility and hatchability rates and the lowest embryo mortality among all treatment groups. Binomial logistic regression confirmed that Se-NP supplementation at both 0.2 mg/kg and 0.4 mg/kg significantly increased the likelihood of reproductive success, whereas SS treatments showed no significant effect. These findings underscore the potential of Se-NPs to improve reproductive outcomes in poultry. Previous studies have shown that Se-NPs enhance fertility by increasing the number of fertile eggs and positively influencing physiological factors such as body weight, sex ratio, age, hormone levels, and environmental conditions [53]. This effect is biologically significant, as selenium is a key trace element incorporated into more than 25 selenoproteins, many of which are essential for antioxidant defense, redox regulation, and endocrine function critical processes for reproductive success [54]. By reducing oxidative damage, Se-NPs help preserve the structural integrity of oocytes and support the function of reproductive hormones such as estrogen and progesterone. This results in improved oocyte quality, proper ovulation, and increased fertility and hatchability [55]. Se-NPs also protect ovarian tissue, support follicular development, and enhance overall ovarian function [23]. Given that oxidative stress is a major limiting factor in avian reproduction, especially under intensive farming conditions, strengthening antioxidant defenses through Se-NP supplementation may extend reproductive lifespan and improve egg viability. Additionally, Se-NPs may alleviate inflammation in the reproductive tissues, thereby supporting better egg quality and reproductive performance [56]. Regarding hatchability, Se-NPs protect developing embryos from oxidative damage during incubation and may support embryonic development by improving antioxidant status [57]. Their role in boosting immune function may further reduce infection-related embryonic losses [58]. Organic selenium sources such as Se-NPs have also been shown to improve sperm storage in the oviduct, extend sperm viability, and increase the proportion of sperm present in the yolk layer [59]. Selenium is equally vital for male reproductive health, playing a key role in testosterone synthesis and maintaining sperm structure. The selenium-containing enzyme glutathione peroxidase (GSH-Px) protects sperm from reactive oxygen species (ROS), thereby improving sperm function and viability. These benefits also extend to females by preserving oocyte integrity and maintaining hormonal stability, reinforcing selenium’s dual role in avian reproduction [60]. The observed reduction in embryo mortality in the Se-NP–treated groups is likely due to their strong antioxidant and anti-inflammatory properties. By enhancing the activity of selenoproteins such as glutathione peroxidase and thioredoxin reductase, Se-NPs effectively neutralize ROS, protect mitochondrial function, and preserve DNA integrity during embryonic development [61]. These mechanisms help maintain a stable intra-egg environment, promoting embryo survival particularly during critical stages of incubation when oxidative stress peaks. Furthermore, the immunomodulatory effects of Se-NPs may reduce infection risk, further decreasing embryonic loss and solidifying the comprehensive reproductive benefits of selenium nanoparticle supplementation [62].

This study also demonstrated that Se-NPs significantly enhanced mating and sexual behaviors in birds, particularly at the 0.4 mg/kg dosage. Poisson regression analysis confirmed significantly higher predicted frequencies of sexual behaviors such as wing flapping, waltzing, and mounting in this group. These improvements are likely due to the antioxidant properties of Se-NPs, which protect reproductive tissues and organs from oxidative stress [63]. Se-NPs influence reproductive behavior by modulating redox-sensitive signaling pathways in both the reproductive organs and the brain. These pathways regulate the release of gonadotropin-releasing hormone (GnRH), which in turn controls luteinizing hormone (LH) and follicle-stimulating hormone (FSH) [62]. In males, Se-NPs enhance sperm quality, motility, and morphology, while in females, they support ovulation and fertility [28]. A key mechanism behind these effects is the incorporation of Se-NPs into selenoproteins such as glutathione peroxidase, thioredoxin reductase, and selenoprotein P. These selenoproteins not only detoxify reactive oxygen species (ROS) but also help maintain cellular energy balance critical for high-energy processes like spermatogenesis and sexual behavior [62]. Specifically, Se-NPs have been shown to enhance the activity of enzymes such as glutathione peroxidase, which neutralize ROS and protect sperm cells from oxidative damage [62]. Se-NPs positively influence male sexual development by reducing oxidative stress, thereby improving sperm motility, enhancing semen quality, and protecting sperm cells [64]. Compared to conventional selenium sources, Se-NPs offer superior bioavailability and cellular uptake. This allows them to more effectively cross physiological barriers, including the blood–testis and blood–brain barriers. Their ability to act on the central nervous system is particularly significant, as sexual behavior is regulated by neuroendocrine pathways involving the hypothalamus, limbic system, and olfactory signals [63]. Unlike traditional selenium supplements, Se-NPs are more effective at stimulating testosterone production a key hormone in male sexual behavior [65]. Selenium also accumulates in the nuclei of reproductive cells, contributing to testosterone synthesis in interstitial cells and supporting the development of germ and Sertoli cells, both essential for testicular function [66,67]. These effects are particularly important with advancing age, emphasizing selenium’s role in testicular maturation and longevity [28]. Additionally, Se-NPs support Leydig cell function, further enhancing testosterone production and sexual behavior [68]. Their ability to cross into central nervous system tissues reinforces their impact on the regulation of sexual behavior [28].

## 5. Conclusions

Supplementation with 0.4 mg/kg Se-NPs significantly enhanced quail productivity and reproductive performance, outperforming SS. This dosage improved HDEP, egg mass, feed conversion ratio, egg quality, fertility, hatchability, and male sexual behavior. These findings highlight the potential of Se-NPs to optimize egg production and reproductive outcomes in poultry, offering a valuable tool for improving flock management. Incorporating Se-NPs at 0.4 mg/kg into quail diets may enhance reproductive efficiency and product quality, providing a promising alternative to conventional selenium sources such as SS. This strategy also supports sustainable poultry production by potentially reducing environmental selenium accumulation. Future research should examine the long-term effects of Se-NP supplementation on quail health and productivity. Investigating optimal dosing, cost-effectiveness, and interactions with other dietary components will be essential. Additionally, studies on the mechanisms behind Se-NPs’ superior bioavailability and tissue deposition compared to SS could inform more effective and targeted supplementation practices.

## Figures and Tables

**Table 1:** Experimental groups and dietary treatments.

Group	Treatment Description	Diet Supplement	Se Source	Se Dose (mg/kg)
1	Control	Basal diet only	–	0
2	Se-NPs Low Dose	Basal diet + Se-NPs	Se-NPs	0.20
3	Se-NPs High Dose	Basal diet + Se-NPs	Se-NPs	0.40
4	Sodium Selenite (SS) Low Dose	Basal diet + SS	SS	0.20
5	Sodium Selenite (SS) High Dose	Basal diet + SS	SS	0.40

**Table 2:** Selenium concentration in feed and excreta and apparent retention index.

Treatment Group	Feed Se (mg/kg DM)	Excreta Se (mg/kg DM)	Apparent Retention Index (%)
Control (basal premix)	0.03	0.02	33.3
Se-NPs (0.2 mg/kg)	0.23	0.08	65.2
Se-NPs (0.4 mg/kg)	0.43	0.15	65.1
SS (0.2 mg/kg)	0.23	0.12	47.8
SS (0.4 mg/kg)	0.43	0.28	34.9

Apparent retention index (%) = (Feed Se − Excreta Se)/Feed Se × 100. This index indicates the fraction of selenium not recovered in excreta; higher values suggest greater retention and bioavailability.

**Table 3:** Major components and nutritional contents of Japanese quail diets.

Ingredients	Contents (%)
Yellow Corn	49.25
Soya bean meal	32.18
Starch	10.15
Limestone	6.50
Di-Calcium Phosphate	1.16
Salt (NaCl)	0.30
Alfalfa leaf powder	0.16
Vitamin and mineral premixture *	0.30
Calculated analysis *	
ME, Kcal/kg	2830
Crude protein	19.63
Crude Fiber	2.21
Ether Extract	2.19
Calcium	2.82
Phosphorous	0.33
Methionine + cystine	0.72
Methionine	0.44
Lysine	1.01

* Each kg of vitamin and mineral mixture included the following: 10,000 IU of retinol, 3500 IU of cholecalciferol, 35 IU of tocopherol, 1.67 mg of phylloquinone, 1.67 mg of thiamine, 2 mg of riboflavin, 3.67 mg of pyridoxine, 0.012 mg of cyanocobalamin, 6.67 mg of pantothenic acid, 16.7 mg of nicotinic acid, 1.67 mg of folic acid, 0.07 mg of biotin, 400 mg of choline chloride, 0.03 mg Selenium, 133.4 g of Mg, 90 mg of Mn, 80 mg of Zn, 25 mg of Fe, 1.67 mg of Cu, and 0.8 mg of I.

**Table 4:** Productive Performance (Mean ± SEM) of Japanese quails supplemented with low and high doses of Se-NPs and SS.

Performance Traits	Weeks	Control	Se-NPs (0.2 mg/kg)	Se-NPs (0.4 mg/kg)	SS (0.2 mg/kg)	SS (0.4 mg/kg)	Total Mean	SEM	*p* Value		
									T	t	T × t
Feed Intake (g)	9th	18.50	19.30	20.00	18.50	19.00	19.06 ^c^	0.10	0.000	0.000	0.007
	11th	21.00	21.50	23.00	21.00	21.17	21.53 ^a^	0.10			
	13th	20.05	20.16	22.43	19.12	20.10	20.37 ^b^	0.10			
	Total Mean	19.85 ^bc^	20.32 ^b^	21.81 ^a^	19.54 ^c^	20.09 ^b^	20.32	0.06			
HDEP %	9th	60.00	68.00	68.00	71.00	70.00	67.40 ^c^	0.19	0.000	0.000	0.000
	11th	70.00	73.00	74.00	73.00	72.00	72.40 ^a^	0.19			
	13th	71.00	70.00	72.00	69.00	65.17	69.43 ^b^	0.19			
	Total Mean	67.00 ^d^	70.33 ^b^	71.33 ^a^	71.00 ^ab^	69.05 ^c^	69.74	0.11			
Egg weight (g)	9th	8.21	10.20	9.00	8.20	7.92	8.70 ^c^	0.02	0.000	0.000	0.000
	11th	8.58	10.61	9.40	8.62	8.68	9.18 ^a^	0.02			
	13th	8.38	10.40	9.20	8.32	8.60	8.98 ^b^	0.02			
	Total Mean	8.40 ^c^	10.40 ^a^	9.20 ^b^	8.38 ^c^	8.40 ^c^	8.95	0.01			
Egg Mass (g)	9th	5.60	6.27	7.40	5.90	5.78	6.19 ^b^	0.01	0.000	0.000	0.000
	11th	5.80	6.53	7.50	6.17	6.00	6.40 ^a^	0.01			
	13th	5.60	6.52	7.30	6.00	5.60	6.20 ^b^	0.01			
	Total Mean	5.67 ^e^	6.44 ^b^	7.40 ^a^	6.02 ^c^	5.79 ^d^	6.26	0.01			
FCR	9th	3.53	3.17	2.94	3.32	3.37	3.26 ^b^	0.01	0.000	0.000	0.000
	11th	3.13	3.00	2.92	3.00	3.30	3.07 ^c^	0.01			
	13th	3.68	3.22	2.97	3.35	3.57	3.35 ^a^	0.01			
	Total Mean	3.45 ^a^	3.13 ^c^	2.94 ^d^	3.22 ^b^	3.41 ^a^	3.23	0.01			

Se-NPs = Selenium Nanoparticles, SS = Sodium selenite, SEM = Standard Error of the Mean, HDEP = Hen Day Egg Production, FCR = Feed Conversion Ratio: The means values with distinct superscripts ^(a–e)^ in a row exhibit a substantial variations at (*p* < 0.05). T = Treatments, t = time, T × t = Treatment × time effects.

**Table 5:** Egg quality parameters (Mean ± SEM) of Japanese quails supplemented with low and high doses of Se-NPs and SS.

Egg Quality	Weeks	Control	Se-NPs (0.2 mg/kg)	Se-NPs (0.4 mg/kg)	SS (0.2 mg/kg)	SS (0.4 mg/kg)	Total Mean	SEM	*p* Value		
									T	T	T × t
Shape Index	9th	66.10	69.94	68.83	65.50	68.00	67.67 ^c^	0.10	0.000	0.000	0.007
	11th	74.83	80.00	72.50	73.00	71.00	74.26 ^a^	0.10			
	13th	63.70	75.00	68.47	66.03	69.20	68.48 ^b^	0.10			
	Total Mean	68.21 ^d^	75.98 ^a^	69.93 ^b^	68.18 ^d^	69.40 ^c^	70.14	0.06			
Shell Ratio	9th	16.00	14.00	18.50	15.50	16.20	16.04 ^b^	0.17	0.000	0.000	0.763
	11th	18.50	16.00	21.20	17.90	18.00	18.32 ^a^	0.17			
	13th	15.00	12.00	17.00	14.00	15.00	14.60 ^c^	0.17			
	Total Mean	16.50 ^b^	14.00 ^c^	18.90 ^a^	15.80 ^b^	16.40 ^b^	16.32	0.10			
Albumen weight (g)	9th	4.50	4.30	3.67	4.10	4.40	4.19 ^b^	0.03	0.000	0.000	0.000
	11th	4.97	4.75	4.17	4.70	4.70	4.65 ^a^	0.03			
	13th	4.00	4.00	3.92	3.20	4.10	3.84 ^c^	0.03			
	Total Mean	4.49 ^a^	4.35 ^a^	3.92 ^b^	4.00 ^b^	4.40 ^a^	4.23	0.02			
Yolk Ratio	9th	35.08	35.33	42.58	35.00	36.55	36.55 ^b^	0.08	0.000	0.000	0.008
	11th	36.42	36.50	43.22	35.33	37.38	37.37 ^a^	0.08			
	13th	34.00	34.00	40.83	34.00	35.25	35.25 ^c^	0.08			
	Total Mean	35.17 ^bc^	35.27 ^b^	42.21 ^a^	35.00 ^c^	34.53 ^c^	36.39	0.05			
Albumen Ratio	9th	31.00	38.00	31.50	35.00	33.00	33.70 ^b^	0.09	0.000	0.000	0.000
	11th	31.42	39.17	32.00	36.00	34.40	34.59 ^a^	0.09			
	13th	30.00	37.00	30.00	33.33	30.83	32.23 ^c^	0.09			
	Total Mean	30.81 ^d^	38.01 ^a^	31.17 ^d^	34.78 ^b^	32.74 ^b^	33.51	0.05			
Yolk Index	9th	36.17	43.13	58.50	45.33	39.50	44.54 ^b^	0.09	0.000	0.000	0.000
	11th	36.25	43.50	59.00	47.00	41.13	45.37 ^a^	0.09			
	13th	35.00	42.00	57.33	44.50	38.20	43.40 ^c^	0.09			
	Total Mean	35.80 ^e^	42.87 ^c^	58.27 ^a^	45.64 ^b^	39.61 ^d^	44.44	0.05			
Albumen Index	9th	7.42	10.10	7.60	7.50	7.30	7.98 ^b^	0.06	0.000	0.000	0.000
	11th	8.50	11.00	7.88	7.70	7.85	8.58 ^a^	0.06			
	13th	7.00	9.42	7.50	7.00	7.17	7.61 ^c^	0.06			
	Total Mean	7.64 ^b^	10.17 ^a^	7.66 ^b^	7.40 ^b^	7.44 ^b^	8.06	0.03			
Haugh Unit	9th	64.60	72.80	74.33	63.00	65.50	68.04 ^b^	0.08	0.000	0.000	0.000
	11th	65.20	73.40	75.30	64.00	67.00	68.98 ^a^	0.08			
	13th	64.00	71.02	74.00	62.00	65.00	67.20 ^c^	0.08			
	Total Mean	64.60 ^d^	72.40 ^b^	74.54 ^a^	63.00 ^e^	65.83 ^c^	68.08	0.04			

Se-NPs = Selenium Nanoparticles, SS = Sodium selenite, SEM = Standard Error of Mean: The means values with distinct superscripts ^(a–e)^ in a row exhibit a substantial variations at (*p* < 0.05). T = Treatments, t = time, T × t = Treatment × time effects.

**Table 6:** Reproductive Performance (Mean ± SEM) of Japanese quails supplemented with low and high doses of Se-NPs and SS.

Variables	Weeks	Control	Se-NPs (0.2 mg/kg)	Se-NPs (0.4 mg/kg)	SS (0.2 mg/kg)	SS (0.4 mg/kg)	Total Mean	SEM	*p* Value		
									T	T	T × t
Fertility %	9th	77.17	80.00	80.83	78.00	76.00	78.40 ^b^	0.17	0.000	0.000	0.199
	11th	80.20	82.00	82.73	81.08	79.50	81.10 ^a^	0.17			
	13th	75.00	78.00	79.00	77.00	75.00	76.80 ^c^	0.17			
	Total Mean	77.46 ^d^	80.00 ^b^	80.86 ^a^	78.69 ^c^	76.83 ^d^	78.77	0.10			
Hatchability %	9th	75.00	80.77	81.94	76.20	74.00	77.58 ^b^	0.09	0.000	0.000	0.000
	11th	76.08	82.58	85.20	78.00	75.10	79.39 ^a^	0.09			
	13th	74.20	79.50	80.00	75.00	72.00	76.14 ^c^	0.09			
	Total Mean	75.09 ^d^	80.95 ^b^	82.38 ^a^	76.40 ^c^	73.70 ^e^	77.71	0.05			
Embryo mortality %	9th	25.00	19.23	18.06	23.80	26.00	22.42 ^b^	0.09	0.000	0.000	0.000
	11th	23.92	17.42	14.80	22.00	24.90	20.61 ^c^	0.09			
	13th	25.80	20.50	20.00	25.00	28.00	23.86 ^a^	0.09			
	Total Mean	24.91 ^b^	19.05 ^d^	17.62 ^e^	23.60	26.30 ^a^	22.29 ^c^	0.05			
Variables	Parameters		B	SE		95% Wald confidence Interval		Wald Chi Square		*p*-value	
						Lower	Upper				
Fertility %	(Intercept)		0.547	1.37		−2.17	3.26	0.16		0.693	
	Se-NPs (0.2 mg/kg)		1.325	0.66		0.37	2.61	4.07		0.044	
	Se-NPs (0.4 mg/kg)		2.093	0.82		0.48	3.71	6.45		0.011	
	SS (0.2 mg/kg)		0.465	0.56		−0.63	1.56	0.69		0.407	
	SS (0.4 mg/kg)		−0.278	0.53		−1.31	0.76	0.28		0.598	
	Control group		0 ^a’^							-	
	Weeks		−2.00	0.12		−0.24	0.23	0.00		1.000	
Hatchability %	(Intercept)		−0.153	1.31		−2.72	2.41	0.01		0.907	
	Se-NPs (0.2 mg/kg)		1.476	0.62		0.28	2.68	5.83		0.016	
	Se-NPs (0.4 mg/kg)		2.064	0.71		−0.67	3.46	8.45		0.004	
	SS (0.2 mg/kg)		0.413	0.53		−0.62	1.45	0.62		0.433	
	SS (0.4 mg/kg)		−0.267	0.52		−1.28	0.74	0.27		0.606	
	Control group		0 ^a’^								
	Weeks		0.026	0.11		−0.19	0.25	0.05		0.819	

Se-NPs = Selenium Nanoparticles, SS = Sodium selenite, SEM = Standard Error of Mean: Values represent the frequency of each behavior observed. The means values with distinct superscripts ^(a–e)^ in a row exhibit a substantial variations at (*p* < 0.05). T = Treatments, t = time, T × t = Treatment × time effects. B = unstandardized regression coefficient, ^a’^. Set to zero because this parameter is redundant.

**Table 7:** Sexual behaviors (Mean ± SEM) of Japanese quails supplemented with low and high doses of Se-NPs and SS.

Sexual Behavior	Weeks	Control	Se-NPs (0.2 mg/kg)	Se-NPs (0.4 mg/kg)	SS (0.2 mg/kg)	SS (0.4 mg/kg)	Total Mean	SEM	*p* Value		
									T	t	T × t
Wing flapping	9th	60.00	80.98	88.50	67.17	61.00	71.53 ^b^	0.20	0.000	0.000	0.004
	11th	62.00	83.00	88.50	68.83	62.00	72.87 ^a^	0.20			
	13th	59.00	80.00	87.50	66.00	62.00	70.90 ^b^	0.20			
	Total Mean	60.33 ^e^	81.33 ^b^	88.17 ^a^	67.33 ^c^	61.67 ^d^	71.77	0.12			
Waltzing	9th	8.00	11.00	13.00	8.00	8.00	9.60 ^b^	0.16	0.000	0.000	0.167
	11th	10.00	12.00	15.00	10.00	9.00	11.20 ^a^	0.16			
	13th	6.00	10.00	12.00	7.00	7.00	8.40 ^c^	0.16			
	Total Mean	8.00 ^c^	11.00 ^b^	13.33 ^a^	8.33 ^c^	8.00 ^c^	9.73	0.10			
Mounting	9th	49.00	65.00	65.83	51.17	50.00	56.20 ^b^	0.12	0.000	0.000	0.124
	11th	50.00	65.83	68.00	53.00	52.00	57.76 ^a^	0.12			
	13th	47.00	62.67	64.83	50.00	48.00	54.66 ^c^	0.12			
	Total Mean	48.67 ^e^	64.50 ^b^	66.22 ^a^	51.39 ^c^	50.28 ^d^	56.21	0.07			
Tidbitting	9th	1.00	3.00	4.00	1.00	0.00	1.8 ^b^	0.07	0.000	0.000	0.000
	11th	2.00	5.00	5.00	1.00	1.00	2.8 ^a^	0.07			
	13th	0.00	2.00	2.00	2.00	3.00	1.8 ^b^	0.07			
	Total Mean	1.00 ^b^	3.33 ^a^	3.67 ^a^	1.33 ^b^	1.33 ^b^	2.13	0.04			
Rear approach	9th	56.00	74.93	77.00	60.00	60.00	65.59 ^c^	0.09	0.000	0.000	0.000
	11th	58.33	78.33	80.83	64.67	60.83	68.60 ^a^	0.09			
	13th	57.00	76.11	77.83	61.00	60.00	66.39 ^b^	0.09			
	Total Mean	57.11 ^e^	76.45 ^b^	78.56 ^a^	61.89 ^c^	60.28 ^d^	66.89	0.05			
Treading	9th	65.00	82.00	86.83	71.00	71.00	75.17 ^b^	0.26	0.000	0.000	0.155
	11th	67.00	84.00	88.00	74.00	72.00	77.00 ^a^	0.26			
	13th	62.00	80.00	86.00	69.00	68.00	73.00 ^c^	0.24			
	Total Mean	64.67 ^d^	82.00 ^b^	86.94 ^a^	71.33 ^c^	70.33 ^c^	75.05	0.12			

Se-NPs = Selenium Nanoparticles, SS = Sodium selenite, SEM= Standard Error of Mean: Values represent the frequency of each behavior observed. The means values with distinct superscripts ^(a–e)^ in a row exhibit a substantial variations at (*p* < 0.05). T = Treatments, t = time, T × t = Treatment × time effects.

**Table 8:** Poisson Regression Analysis of the Impact of Se-NPs and SS Supplementation on Male Sexual Behavior in Quails.

Sexual Behaviors	Groups	B (Estimate)	SE	95% Wald Confidence Interval		*p*-Value	Exp (B)	95% Wald Confidence Interval for Exp (B)	
				Lower	Upper			Lower	Upper
Wing Flapping	(Intercept)	4.12	0.02	4.06	4.18	0.000	61.80	58.31	65.51
	Se-NPs (0.2 mg/kg)	0.29	0.01	0.27	0.32	0.000	1.34	1.31	1.38
	Se-NPs (0.4 mg/kg)	0.37	0.01	0.35	0.41	0.000	1.46	1.42	1.49
	SS (0.2 mg/kg)	0.11	0.01	0.08	0.13	0.003	1.11	1.08	1.14
	SS (0.4 mg/kg)	0.02	0.01	−0.01	0.05	0.124	1.02	0.99	1.05
	Control group	0 ^a^					1		
	Weeks	−0.00	0.00	−0.007	0.00	0.388	0.99	0.99	1.00
Waltzing	(Intercept)	2.41	0.08	2.26	2.57	0.000	11.21	9.58	13.13
	Se-NPs (0.2 mg/kg)	0.31	0.03	0.24	0.39	0.000	1.37	1.28	1.47
	Se-NPs (0.4 mg/kg)	0.51	0.03	0.44	0.58	0.000	1.66	1.55	1.78
	SS (0.2 mg/kg)	0.04	0.03	−0.03	0.11	0.294	1.04	0.96	1.12
	SS (0.4 mg/kg)	−9.27	0.03	−0.07	0.07	1.000	1.00	0.92	1.08
	Control group	0 ^a^					1		
	Weeks	−0.03	0.00	−0.04	−0.01	0.000	0.97	0.95	0.98
Mounting	(Intercept)	3.96	0.03	3.89	4.02	0.000	52.45	49.12	56.01
	Se-NPs (0.2 mg/kg)	0.28	0.01	0.25	0.31	0.000	1.32	1.28	1.36
	Se-NPs (0.4 mg/kg)	0.30	0.01	0.27	0.33	0.000	1.36	1.32	1.40
	SS (0.2 mg/kg)	0.05	0.01	0.02	0.08	0.001	1.05	1.02	1.08
	SS (0.4 mg/kg)	0.03	0.01	0.00	0.06	0.039	1.03	1.00	1.06
	Control group	0 ^a^					1		
	Weeks	−0.00	0.00	−0.01	−0.00	0.017	0.99	0.98	0.99
Tidbitting	(Intercept)	−2.48	0.18	−0.35	0.35	1.000	1.00	0.70	1.42
	Se-NPs (0.2 mg/kg)	1.20	0.08	1.02	1.38	0.000	3.33	2.79	3.97
	Se-NPs (0.4 mg/kg)	1.29	0.08	1.12	1.47	0.000	3.66	3.08	4.36
	SS (0.2 mg/kg)	0.28	0.10	0.08	0.49	0.006	1.33	1.08	1.63
	SS (0.4 mg/kg)	0.28	0.10	0.08	0.49	0.006	1.33	1.08	1.63
	Control group	0 ^a^					1		
	Weeks	2.258 × 10^−16^	0.01	−0.02	0.02	1.000	1.00	0.97	1.02
Rear approach	(Intercept)	3.92	0.03	3.86	3.98	0.000	50.44	47.48	53.58
	Se-NPs (0.2 mg/kg)	0.29	0.01	0.26	0.31	0.000	1.33	1.30	1.37
	Se-NPs (0.4 mg/kg)	0.31	0.01	0.29	0.34	0.000	1.37	1.33	1.41
	SS (0.2 mg/kg)	0.08	0.01	0.05	0.10	0.000	1.08	1.05	1.11
	SS (0.4 mg/kg)	0.05	0.01	0.02	0.08	0.000	1.05	1.02	1.08
	Control group	0 ^a^					1		
	Weeks	0.01	0.00	0.00	0.01	0.000	1.01	1.00	1.01
Treading	(Intercept)	4.24	0.02	4.19	4.30	0.000	70.00	66.14	74.09
	Se-NPs (0.2 mg/kg)	0.23	0.01	0.21	0.26	0.000	1.26	1.23	1.30
	Se-NPs (0.4 mg/kg)	0.29	0.01	0.27	0.32	0.000	1.34	1.31	1.37
	SS (0.2 mg/kg)	0.09	0.01	0.07	0.12	0.000	1.10	1.07	1.13
	SS (0.4 mg/kg)	0.08	0.01	0.05	0.11	0.000	1.08	1.05	1.11
	Control group	0 ^a^					1		
	Weeks	−0.00	0.00	−0.01	−0.00	0.004	0.99	0.98	0.99

Se-NPs = Selenium Nanoparticles, SS = Sodium selenite, SE = Standard Error: ^a^. set to zero because this parameter is redundant to zero.

## Data Availability

The data presented in this study are available on request from the corresponding author.

## References

[1] Bagh J., Panigrahi B., Panda N., Pradhan C.R., Mallik B.K., Majhi B., Rout S.S. (2016). Body weight, egg production, and egg quality traits of gray, brown, and white varieties of Japanese quail (*Coturnix coturnix japonica*) in coastal climatic condition of Odisha. Vet. World.

[2] Khan S.H., Naseer J., Tahir M.N., Anjum K., Khan M.H., Nadeem A., Latif A. (2023). Evaluating the effects of processed pigeon pea seed meal on growth performance and digestibility in Japanese quails (*Coturnix japonica*). Trends Anim. Poult. Sci..

[3] Al-Khalaifah H., Naz S., Al-Atiyat R., Khan R.U., Abudabos A., Alhidary I.A. (2025). Eco-Friendly Selenium nanoparticles from Capsicum annuum: Impact on growth efficiency, blood biochemistry, immune response, intestinal morphology, and profitability in broiler chickens. Poult. Sci..

[4] Huang M.Y., An Y.C., Zhang S.Y., Qiu S.J., Yang Y.Y., Liu W.C. (2024). Metabolomic analysis reveals biogenic selenium nanoparticles improve the meat quality of thigh muscle in heat-stressed broilers is related to the regulation of ferroptosis pathway. Poult. Sci..

[5] Surai P.F., Kochish I.I., Fisinin V.I., Velichko O.A. (2018). Selenium in poultry nutrition: From sodium selenite to organic selenium sources. J. Poult. Sci..

[6] Ye X.Q., Zhu Y.R., Yang Y.Y., Qiu S.J., Liu W.C. (2023). Biogenic selenium nanoparticles synthesized with alginate oligosaccharides alleviate heat stress-induced oxidative damage to organs in broilers through activating Nrf2-mediated anti-oxidation and anti-ferroptosis pathways. Antioxidants.

[7] Nabi F., Arain M.A., Hassan F., Umar M., Rajput N., Alagawany M., Syed S.F., Soomro J., Somroo F., Liu J. (2020). Nutraceutical role of selenium nanoparticles in poultry nutrition: A review. J. World’s Poult. Sci..

[8] Yang Y.Y., An Y.C., Zhang S.Y., Huang M.Y., Ye X.Q., Zhao Z.H., Liu W.C. (2023). Biogenic selenium nanoparticles synthesized using alginate oligosaccharides attenuate heat stress-induced impairment of breast meat quality via regulating oxidative stress, metabolome and ferroptosis in broilers. Antioxidants.

[9] Abudabos A.M., Al-Atiyat R.M., Stanley D., Aljassim R., Albatshan H.A. (2017). The effect of corn distiller’s dried grains with solubles (DDGS) fortified with enzyme on growth performance of broiler. Environ. Sci. Pollut. Res. Int..

[10] Chand N., Naz S., Irfan M., Khan R.U., Rehman Z.U. (2018). Effect of sea buckthorn (*Hippophae rhamnoides* L.) seed supplementation on egg quality and cholesterol of Rhode Island Red × Fayoumi laying hens. Korean J. Food Sci. Anim. Resour..

[11] Rahman Z., Naz S., Khan R.U., Tahir M. (2017). An update on the potential application of L-carnitine in poultry. World’s Poult. Sci. J..

[12] Swain P.S., Prusty S., Rao S.B.N., Rajendran D., Patra A.K. (2021). Essential nanominerals and other nanomaterials in poultry nutrition and production. Advances in Poultry Nutrition Research.

[13] Çiçek S., Özoğul F. (2021). Effects of selenium nanoparticles on growth performance, hematological, serum biochemical parameters, and antioxidant status in fish. Anim. Feed Sci. Technol..

[14] Naz S., Bibi G., Nadeem R., Alhidary I., Dai S., Israr M., Khan R.U. (2024). Evaluation of biological selenium nanoparticles on growth performance, histopathology of vital organs and genotoxicity in Japanese quails (*Coturnix coturnix japonica*). Vet. Q..

[15] Zhou W., Miao S., Zhu M., Dong X., Zou X. (2021). Effect of glycine nano-selenium supplementation on production performance, egg quality, serum biochemistry, oxidative status, and intestinal morphology in laying hens. Biol. Trace Elem. Res..

[16] Li J., Sun K., Ni L., Wang X., Wang D., Zhang J. (2012). Sodium selenosulfate at an innocuous dose markedly prevents cisplatin-induced gastrointestinal toxicity. Toxicol. Appl. Pharmacol..

[17] Ali A.A., Soliman E.S., Hamad R.T., El-Borad O.M., Hassan R.A., Helal M.S. (2020). Preventive, behavioral, productive, and tissue modification using green synthesized selenium nanoparticles in the drinking water of two broiler breeds under microbial stress. Braz. J. Poult. Sci..

[18] Reda F.M., El-Saadony M.T., Elnesr S.S., Alagawany M., Tufarelli V. (2020). Effect of dietary supplementation of biological curcumin nanoparticles on growth and carcass traits, antioxidant status, immunity and caecal microbiota of Japanese quails. Animals.

[19] Mahmoud R., Salama B., Safhi F.A., Pet I., Pet E., Ateya A. (2024). Assessing the impacts of different levels of nano-selenium on growth performance, serum metabolites, and gene expression in heat-stressed growing quails. Vet. Sci..

[20] Batool S., Khan M.A., Naz S., Shah M., Alrefai A.F., Ibiwoye D.I., Momand N.K., Khan R.U. (2025). Anticoccidial effect of bitter apple (*Citrullus colocynthis*) seed powder and organic selenium nanoparticles in mitigating Eimeria tenellainduced challenge in quails. J. Appl. Anim. Res..

[21] Khan R.U., Al-Khalaifah H., Usama M., Naz S., Khan S., Batool S., Alrefaei A.F., Konca Y., Abdelrahman S., Selvaggi M. (2025). Effect of dietary supplementation of biosynthesized nano-selenium particles on growth, blood indices, antioxidant status, immune response and histological features of intestine in broilers. Food Agric. Immunol..

[22] Elnaggar A.S., Ghazalah A., Elsayed A.H., Abdelalem A. (2020). Impact of selenium sources on productive and physiological performance of broilers. Egypt. Poult. Sci. J..

[23] Rana T. (2021). Nano-selenium on reproduction and immunocompetence: An emerging progress and prospect in the productivity of poultry research. Trop. Anim. Health Prod..

[24] El-Kazaz S.E., Abo-Samaha M.I., Hafez M.H., El-Shobokshy S.A., Wirtu G. (2020). Dietary supplementation of nano-selenium improves reproductive performance, sexual behavior and deposition of selenium in the testis and ovary of Japanese quail. J. Adv. Vet. Anim. Res..

[25] Meng T., Liu Y.L., Xie C.Y., Zhang B., Huang Y.Q., Zhang Y.W., Wu X. (2019). Effects of different selenium sources on laying performance, egg selenium concentration, and antioxidant capacity in laying hens. Biol. Trace Elem. Res..

[26] Shokraneh M., Sadeghi A.A., Mousavi S.N., Esmaeilkhanian S., Chamani M. (2020). Effects of in ovo injection of nano-selenium and nano-zinc oxide and high eggshell temperature during late incubation on antioxidant activity, thyroid and glucocorticoid hormones and some blood metabolites in broiler hatchlings. Acta Sci. Anim. Sci..

[27] Khalil W.A., El-Harairy M.A., Zeidan A.E., Hassan M.A. (2019). Impact of selenium nanoparticles in semen extender on bull sperm quality after cryopreservation. Theriogenology.

[28] Salimi T., Hajarian H., Karamishabankareh H., Soltani L. (2024). Effects of sodium selenite, cysteamine, bacterially synthesized Se-NPs, and cysteamine loaded on Se-NPs on ram sperm cryopreservation. Sci. Rep..

[29] Nassef E., Saker O., Shukry M. (2020). Effect of Se sources and concentrations on performance, antioxidant defense, and functional egg quality of laying Japanese quail (*Coturnix japonica*). Environ. Sci. Pollut. Res..

[30] Lukanov H., Pavlova I., Genchev A. (2020). Effect of the quail’s productive type on the incubation characteristics of domestic quail eggs (*Coturnix japonica domestica*). Bulg. J. Agric. Sci..

[31] Randal M., Bolla G. (2008). Raising Japanese quail. Primefacts.

[32] Bean T.G., Beasley V.R., Berny P., Eisenreich K.M., Elliott J.E., Eng M.L., Rattner B.A. (2024). Toxicological effects assessment for wildlife in the 21st century: Review of current methods and recommendations for a path forward. Integr. Environ. Assess. Manag..

[33] NRC (National Research Council) (1994). Nutrient Requirements of Poultry.

[34] Batra G., Gortzi O., Lalas S.I., Galidi A., Alibade A., Nanos G.D. (2017). Enhanced antioxidant activity of Capsicum annuum, L. and *Moringa oleifera* L. extracts after encapsulation in microemulsions. Chem. Eng..

[35] Sarmiento-García A., Sevim B., Olgun O., Ahmet-Gökmen S. (2022). Effects of different inorganic selenium levels in laying quails (*Coturnix coturnix japonica*) diets on performance, egg quality, and serum biochemical parameters. Vet. México OA.

[36] Khan M.M., Hossain M.N., Baset M.A., Uddin M.N. (2018). Effect of organic selenium supplementation on productive and reproductive performances of Japanese quails. J. Sylhet Agric. Univ..

[37] Elkhateeb F.S., Ghazalah A.A., Lohakare J., Abdel-Wareth A.A. (2024). Selenium nanoparticle inclusion in broiler diets for enhancing sustainable production and health. Sci. Rep..

[38] Alagawany M., Qattan S.Y., Attia Y.A., El-Saadony M.T., Elnesr S.S., Mahmoud M.A., Reda F.M. (2021). Use of chemical nano-selenium as an antibacterial and antifungal agent in quail diets and its effect on growth, carcasses, antioxidant, immunity, and caecal microbes. Animals.

[39] Zakaria A.H., Omar O.H. (2013). Egg laying pattern, egg weight, body weight at hatch, and sex ratio bias relative to oviposition time of young- and mid-age broiler breeders. Anim. Reprod. Sci..

[40] Elfiky A.A., Enab A.A., Zanaty G.A., Morsy A.S., Sewalem H.Z. (2021). Productive performance and egg quality traits of laying hens fed on diets treated with nano-selenium under hot desert conditions. Menoufia J. Anim. Poult. Fish Prod..

[41] Köhrle J., Frädrich C. (2022). Deiodinases control local cellular and systemic thyroid hormone availability. Free Radic. Biol. Med..

[42] Mojadadi A., Au A., Salah W., Witting P., Ahmad G. (2021). Role for selenium in metabolic homeostasis and human reproduction. Nutrients.

[43] England A.D., Gharib-Naseri K., Kheravii S.K., Wu S.B. (2022). Rearing broilers as mixed or single-sex: Relevance to performance, coefficient of variation, and flock uniformity. Poult. Sci..

[44] Mengistu S.B., Mulder H.A., Benzie J.A., Komen H. (2020). A systematic literature review of the major factors causing yield gap by affecting growth, feed conversion ratio, and survival in Nile tilapia (*Oreochromis niloticus*). Rev. Aquac..

[45] Duman M., Şekeroğlu A., Yıldırım A., Eleroğlu H.A., Camcı Ö. (2016). Relation between egg shape index and egg quality characteristics. Eur. Poult. Sci..

[46] Drabik K., Batkowska J., Vasiuk K., Pluta A. (2020). The impact of eggshell colour on the quality of table and hatching eggs derived from Japanese Quail. Animals.

[47] Orellana Galindo L. (2023). Effect of Eggshell Translucency and Color on Broiler Egg Hatchability and Chick Quality and Its Relationship with Other Eggshell Quality Parameters. Master’s Thesis.

[48] Patra A., Lalhriatpuii M. (2020). Progress and prospect of essential mineral nanoparticles in poultry nutrition and feeding—A review. Biol. Trace Elem. Res..

[49] Alig B.N., Malheiros R.D., Anderson K.E. (2023). Evaluation of physical egg quality parameters of commercial brown laying hens housed in five production systems. Animals.

[50] Satti S., Naz S., Khan R.U., Alrefaei A.F., Almutairi M., Momand N.K., Ibiwoye D.I. (2025). Comparative effects of selenium nanoparticles and sodium selenite on selenium bioaccumulation in quail tissues and its transfer to progeny. J. Appl. Anim. Res..

[51] Mohammadsadeghi F., Afsharmanesh M., Salarmoini M., Bami M.K. (2023). The effect of replacing sodium selenite with selenium-chitosan in laying hens on production performance, egg quality, egg selenium concentration, microbial population, immunological response, antioxidant enzymes, and fatty acid composition. Poult. Sci..

[52] Urso U.R., Dahlke F., Maiorka A., Bueno I.J.M., Schneider A.F., Surek D., Rocha C. (2015). Vitamin E and selenium in broiler breeder diets: Effect on live performance, hatching process, and chick quality. Poult. Sci..

[53] Surai P.F., Fisinin V.I. (2014). Selenium in poultry breeder nutrition. An update. Anim. Feed Sci. Technol..

[54] Zhang F., Li X., Wei Y. (2023). Selenium and selenoproteins in health. Biomolecules.

[55] Shalaby O.E., Ahmed Y.H., Mekkawy A.M., Mahmoud M.Y., Elbargeesy G.A. (2025). The ameliorative effect of selenium-loaded chitosan nanoparticles against silver nanoparticles-induced ovarian toxicity in female albino rats. J. Ovarian Res..

[56] Muthukumaran D., Shanmugam R. (2024). Nanoparticle-based interventions for polycystic ovary syndrome: A review of mechanisms and therapeutic potential. J. Drug Deliv. Sci. Technol..

[57] Mathur P., Jha S., Ramteke S., Jain N.K. (2018). Pharmaceutical aspects of silver nanoparticles. Artif. Cells Nanomed. Biotechnol..

[58] Unterweger H., Lyer S., Janko C., Friedrich R.P., Cicha I., Tietze R., Alexiou C. (2020). Nanomedicine for infectious diseases. Nanomedicine.

[59] Zubair M., Martyniuk C.J., Partyka A., Saleemi M.K. (2023). Dietary use of selenium: A review of the antioxidant and scavenging effects on the poultry male reproductive system. World’s Poult. Sci. J..

[60] Qazi I.H., Angel C., Yang H., Zoidis E., Pan B., Wu Z., Ming Z., Zeng C.J., Meng Q., Han H. (2019). Role of selenium and selenoproteins in male reproductive function: A review of past and present evidence. Antioxidants.

[61] Patel K.D., Keskin-Erdogan Z., Sawadkar P., Sharifulden N.S.A.N., Shannon M.R., Patel M., Kim H.W. (2024). Oxidative stress modulating nanomaterials and their biochemical roles in nanomedicine. Nanoscale Horiz..

[62] Yuan S., Zhang Y., Dong P.Y., Yan Y.M.C., Liu J., Zhang B.Q., Zhang X.F. (2024). A comprehensive review on potential role of selenium, selenoproteins, and selenium nanoparticles in male fertility. Heliyon.

[63] Zambonino M.C., Quizhpe E.M., Mouheb L., Rahman A., Agathos S.N., Dahoumane S.A. (2023). Biogenic selenium nanoparticles in biomedical sciences: Properties, current trends, novel opportunities, and emerging challenges in theranostic nanomedicine. Nanomaterials.

[64] Zhou C., Zhang H., Wu Y., Ahmed N. (2023). Effect of nano-selenium on exosomes secretion associated with sperm maturation within the epididymis. Micron.

[65] Khalaf A.A., Ahmed W.M.S., Moselhy W.A., Abdel-Halim B.R., Ibrahim M.A. (2019). Protective effects of selenium and nano-selenium on bisphenol-induced reproductive toxicity in male rats. Hum. Exp. Toxicol..

[66] Jerysz A., Lukaszewicz E. (2013). Effect of dietary selenium and vitamin E on ganders’ response to semen collection and ejaculate characteristics. Biol. Trace Elem. Res..

[67] Mahmoud G.B., Abdel-Raheem S.M., Hussein H.A. (2013). Effect of combination of vitamin E and selenium injections on reproductive performance and blood parameters of Ossimi rams. Small Rumin. Res..

[68] Alrashidi M., Gomaa H. (2023). The protective effect of selenium nanoparticles against mono-sodium glutamate-induced alterations in male albino rats: The effect of nano-selenium against MSG-toxicity. J. Qassim Univ. Sci..

